# Enhancing Spatiotemporal Resolution of MCCA SMAP Soil Moisture Products over China: A Comparative Study of Machine Learning-Based Downscaling Approaches

**DOI:** 10.3390/s26041383

**Published:** 2026-02-22

**Authors:** Zhuoer Ma, Peng Chen, Hao Chen, Hang Liu, Yuchen Zhang, Binyi Huang, Yang Hong, Shizheng Sun

**Affiliations:** 1 College of Geomatics, Xi’an University of Science and Technology, Xi’an 710054, China; 2 State Key Laboratory of Information Engineering in Surveying, Mapping and Remote Sensing, Wuhan University, Wuhan 430079, China; 3 School of Geodesy and Geomatics, Wuhan University, Wuhan 430079, China

**Keywords:** soil moisture downscaling, MCCA SMAP, machine learning, China

## Abstract

As a key parameter of the Earth’s ecosystem, soil moisture significantly influences land-atmosphere interactions and has important applications in meteorology, hydrology, and agricultural studies. However, existing passive microwave remote sensing products of soil moisture are limited by their discontinuous temporal coverage and relatively coarse spatial resolution (typically 25–55 km), which cannot meet the requirements for fine-scale applications. This study developed and compared four machine learning-based downscaling approaches to improve the spatiotemporal resolution of MCCA SMAP soil moisture products. The methodology involved establishing complex nonlinear relationships between soil moisture and various high-resolution surface parameters including albedo, evapotranspiration, precipitation, and soil properties. High-resolution soil moisture maps were generated by leveraging the scale-invariant characteristics between soil moisture and surface parameters, followed by comprehensive evaluation using in situ ground observations and triple collocation analysis. The results demonstrated that all downscaling models showed excellent consistency with original MCCA SMAP observations (R > 0.93, RMSE < 0.033 m^3^ m^−3^), while successfully providing enhanced spatial details. The Random Forest (RF) model exhibited superior performance, showing higher correlation coefficients and lower biases when compared with in situ measurements. Uncertainty analysis revealed relatively low uncertainty levels for all models except Backpropagation Neural Network (BPNN) model. The RF-downscaled products accurately tracked temporal variations of soil moisture and showed good responsiveness to precipitation patterns, demonstrating their potential for fine-scale hydrological applications and regional environmental monitoring.

## 1. Introduction

Soil Moisture (SM) is a key variable in the hydrological system, regulating the exchange of matter and energy between the land surface and the atmosphere. It holds significant importance in Earth science research and global climate change monitoring [1,2,3]. Developing long-term, high-accuracy SM datasets with high spatial and temporal resolution is crucial for a variety of Earth system applications, such as flood and drought monitoring [4,5], hydrological and climate modeling and land surface simulation [6,7], crop yield estimation and food security monitoring [8,9], and climate change studies [10,11].

Currently, the primary methods for acquiring SM data fall into three categories: ground-based observations, model data assimilation, and microwave remote sensing [12]. Various techniques have been developed and applied for point-scale measurement of surface SM using ground instruments, such as neutron probe (NP) [13], time domain reflectometry (TDR) [14], and the ECH_2_O probe model EC-5 sensor [15], etc. Although ground-based measurements can provide highly accurate monitoring of SM across different soil layers [16], due to the sparse distribution of observation sites and the spatial heterogeneity of terrain, land cover, soil properties, and precipitation, point measurements often fail to accurately capture the spatiotemporal characteristics of regional SM [17]. Therefore, obtaining real-time SM information at regional to global scales via ground observation networks remains a major challenge [18]. Moreover, the high maintenance cost of soil moisture sensors and associated equipment limits their widespread use [12]. These limitations make it difficult for ground observations alone to meet the requirement for large-scale and spatially continuous soil moisture monitoring. Spatiotemporally continuous SM products can also be obtained through Land Surface Models (LSMs) or global reanalysis systems. For instance, the Global Land Data Assimilation System (GLDAS) provides global SM products by integrating multi-source observational data [19], and the ERA5-Land reanalysis dataset generates global SM data at a daily scale and 0.1° spatial resolution [20]. However, these results exhibit significant model dependency—meaning that even when using the same meteorological inputs, different LSMs can produce markedly different SM outputs, and this inconsistency is commonly observed across various reanalysis products and model outputs [21]. This model dependency introduces considerable uncertainty, which motivates the development of alternative approaches to obtain more reliable soil moisture information.

Over the past few decades, satellite remote sensing—especially microwave remote sensing—has become a key method for obtaining SM, thanks to its wide coverage, all-weather capability, and high observation frequency [22,23]. To date, numerous satellite platforms equipped with various active and passive microwave sensors have provided soil moisture observation products, such as Soil Moisture Active Passive (SMAP) [24], Soil Moisture and Ocean Salinity (SMOS) [25], the Aqua satellite, equipped with the Advanced Microwave Scanning Radiometer–EOS (AMSR-E) [26], Global Change Observation Mission–Water (GCOM-W), equipped with the Advanced Microwave Scanning Radiometer 2 (AMSR2) [27], Satélite de Aplicaciones Científicas–D (SAC-D), equipped with the Aquarius sensor [28], Cyclone Global Navigation Satellite System (CYGNSS) [29] and FY-3B [30]. Although the accuracy of satellite-derived SM products has continuously improved, their temporal resolution (1–2 days) and spatial resolution (approximately 25–50 km) are still inferior to ground-based observations due to limitations imposed by the aperture size of radiometer antennas [31]. This limits their applicability in high-precision hydrological, agricultural, and Earth science studies at finer spatial scales [32]. To address this limitation, researchers have developed various techniques aimed at enhancing the spatial resolution of SM data across different scales. Based on the types of input data and computational approaches, these downscaling algorithms can be broadly classified into three categories [33]: (1) Satellite-based methods, including active–passive microwave data fusion [34,35,36] and optical/thermal–microwave fusion approaches. Specifically, the National Aeronautics and Space Administration (NASA) has developed a 3 km downscaled product using an active–passive downscaling algorithm that combines SMAP and Sentinel-1 data, which performs well at both 1 km and 3 km resolutions [34]. Montzka et al. directly decoupled radiometer-based SM products from radar-based SM products and proposed a method that directly fuses passive/active SM products with a wavelet-based image enhancement technique [35]. Peng et al. applied a triple collocation and least squares fusion approach to integrate multi-source satellite data and produced a 12.5 km downscaled SM product [36]. In addition, due to the ability of optical/thermal infrared remote sensing to provide higher-resolution surface parameters, many studies have attempted to use such data to construct downscaling factors for optimizing microwave SM products [33,37,38,39]. However, optical data are vulnerable to cloud cover, vegetation, and swath width limitations, and the assumption of simple linear scale transformation may not be broadly applicable over large areas [40,41]. (2) Model-based methods, including approaches based on statistical models and those based on hydrological or land surface process models. The former describe and preserve the statistical characteristics of soil moisture across different scales through techniques such as geostatistics, wavelets [42], and multifractals [43,44], thereby enabling scale conversion. The latter integrate soil properties and physical processes into hydrological/LSMs and can be combined with remote sensing observations to achieve fine-scale soil moisture estimation through parameter optimization (deterministic downscaling) [45], statistical regression (statistical downscaling) [46], or data assimilation (dynamic downscaling) [47]. (3) Methods based on geoinformation data, which utilize the relationships between soil moisture and other high-resolution land surface parameters related to SM [48,49,50]. Specifically, Fu et al. applied multiple linear regression (MLR) and random forest (RF) to downscale the SMAP L4 SM data over the Yellow River source region, incorporating surface moving average variation differences during freeze-thaw periods [49]. This resulted in a 1 km SM dataset spanning 2015–2021, with a temporal resolution of twice daily. Karthikeyan and Mishra used extreme gradient boosting (XGBoost) to produce a 1 km daily SM dataset for the U.S. from 2015 to 2019, with validation showing it could effectively capture the spatiotemporal variability of SM [51]. However, in recent years, such purely data-driven retrieval approaches have faced criticism due to their lack of physical interpretability and generalization capabilities. Some studies have begun integrating machine learning with physically based inversion models. For instance, Li et al. implemented a differentiable water-cloud model (WCM) on a machine learning platform and combined it with a neural network to retrieve soil moisture at a 10 m resolution [52]. While physically based models offer strong interpretability and generalizability, their performance is often limited under complex surface conditions due to simplified assumptions and data constraints. Integrating multi-source data with machine learning methods can help overcome these limitations and improve the accuracy and resolution of SM products.

The limitations of previous downscaling studies are mainly reflected in two aspects. First, traditional downscaling research has largely focused on pixel-to-pixel regression models that neglect spatial neighborhood effects, reducing accuracy in regions with complex terrain. Second, most previous studies simplify the problem as a static mapping, ignoring temporal dependencies and past hydrometeorological conditions that influence soil moisture dynamics.

The complex terrain and climate of China result in significant spatial and temporal heterogeneity in soil moisture. However, current satellite products such as SMAP (9–36 km resolution) struggle to capture fine-scale hydrological patterns over heterogeneous surfaces, including mountain–plain transitions, agro-pastoral zones, and urban clusters. The lack of high-resolution (≤1 km) SM data limits research on regional water cycles and drought monitoring. To address this, this study downscales 2019 MCCA SMAP data over China to 1 km by integrating multi-source variables using four machine learning models: RF, Backpropagation Neural Network (BPNN), Convolutional Neural Network–Gated Recurrent Unit (CNN–GRU), and Residual Neural Network (ResNet). Model performance and uncertainty are evaluated, and the best-performing product is validated against in situ data. Differences from existing products and spatial relationships between SM and key variables are also examined. The paper is structured as follows: Section 2 describes the study area and datasets; Section 3 details the downscaling methods; Section 4 presents experimental results; Section 5 discusses the findings; and Section 6 concludes the study.

## 2. Study Area and Data

**Figure 1:**
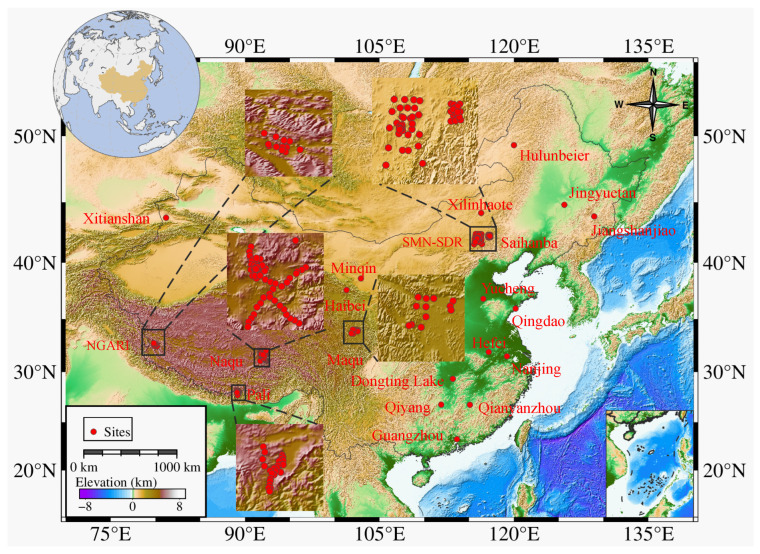
Topographic map of the study area. The red circles indicate the locations of ground observation stations.

This study focuses on the Chinese mainland, located in the southeastern part of the Eurasian continent. The region is influenced by complex topography and atmospheric circulation, resulting in a wide variety of climate types, including monsoon climate, temperate continental climate, and the alpine climate unique to the Tibetan Plateau. These climatic differences significantly impact regional hydrological processes. Additionally, China features diverse land cover types such as wetlands, forests, and urban areas, each of which affects soil water retention to varying degrees. Under the combined influence of these factors, soil moisture across the study area exhibits pronounced spatiotemporal heterogeneity, which challenges the assumptions of spatial stationarity and scale invariance underlying conventional downscaling approaches. Consequently, China provides an ideal testing ground for evaluating the ability of machine learning-based downscaling methods to capture nonlinear relationships and spatial variability under highly heterogeneous environmental conditions, as well as for systematically assessing the accuracy of different downscaling approaches. Figure 1, produced using the Generic Mapping Tools (GMT), illustrates the topographic features of the study area and the spatial distribution of ground observation stations.

This study utilizes four main categories of data to generate high spatiotemporal resolution downscaled SM products: (1) satellite-based products, including: Moderate Resolution Imaging Spectroradiometer (MODIS), SMAP, and Global Precipitation Measurement (GPM) data; (2) model-based products, including: ERA5-Land data, Global Land Evaporation Amsterdam Model (GLEAM) data; (3) in situ soil moisture data; and (4) ancillary data, including Shuttle Radar Topographic Mission (SRTM) Digital Elevation Model (DEM) data and soil texture data from the Harmonized World Soil Database (HWSD). Basic information for each dataset is summarized in Table 1, with further details provided below.

### 2.1. Satellite-Based Products

MODIS sensors aboard Terra and Aqua satellites provide near-daily global optical data. The high-resolution optical auxiliary products used in this study include the Normalized Difference Vegetation Index (NDVI) from MOD13A2, Land Cover Type (LCT) from MCD12Q1, and surface albedo consisting of Black-Sky Albedo (BSA) and White-Sky Albedo (WSA) from MCD43A3. Specifically, MCD43A3 provides black-sky and white-sky albedo data at 500 m daily resolution across different spectral bands; MOD13A2 is a gridded Level-3 product with 1 km spatial and 16-day temporal resolution; MCD12Q1 offers global land cover data at 500 m annual resolution from 2001 to present.

Launched by NASA on January 31, 2015, the SMAP satellite is the second L-band soil moisture monitoring satellite following SMOS. Its uniqueness lies in the combined use of an L-band passive microwave radiometer (1.41 GHz, ∼40 km resolution) and an active synthetic aperture radar (1.26 GHz, ∼3 km resolution) for global soil moisture observation. However, in July of the same year, the active radar malfunctioned and was permanently decommissioned, leaving the SMAP mission to rely primarily on the passive microwave radiometer. Considering that SMAP’s official retrievals are constrained by MODIS vegetation indices, which may not be suitable for further downscaling using the same inputs, this study adopts the SMAP soil moisture and vegetation optical depth product based on the Multi-Channel Collaborative Algorithm (MCCA) provided by the National Tibetan Plateau/Third Pole Environment Data Center (http://data.tpdc.ac.cn). Unlike the official SMAP products, MCCA does not rely on external vegetation parameters (e.g., MODIS NDVI) but jointly retrieves soil moisture and vegetation optical depth using analytical relations among brightness temperatures and self-constraints among surface parameters. Therefore, using this product addresses the potential limitations of employing MODIS vegetation indices again for downscaling. It takes L-band H-polarized brightness temperature as the core channel (CC) and uses other channels for auxiliary constraints, improving both accuracy and general applicability. Validation against 19 dense in situ networks worldwide, including SMAP core and independent sites, shows that MCCA soil moisture generally achieves lower ubRMSE than SMAP products, indicating greater stability and reliability [62,63]. In this study, high-quality retrievals were selected based on retrieval quality flags, and data from both ascending and descending orbits were aggregated to enhance sampling density.

GPM, jointly launched by NASA and Japan Aerospace Exploration Agency (JAXA) on 27 February 2014, is an international satellite network providing global precipitation observations using microwave radiometers and radar. GPM precipitation products are valuable for research in water resource management and natural disaster early warning. The GPM IMERG Final Precipitation product integrates multi-source satellite observations and in situ data, with rigorous calibration, offering precipitation estimates at 0.1° spatial resolution across multiple temporal scales. This study uses the daily precipitation product. More information is available at GPM (https://gpm.nasa.gov/).

### 2.2. Model-Based Products

The ERA5-Land reanalysis dataset, developed by the Copernicus Climate Change Service (C3S) under the European Centre for Medium-Range Weather Forecasts (ECMWF), is the fifth-generation atmospheric reanalysis product. Compared to ERA5 and ERA5-Interim, ERA5-Land offers hourly land surface variables from 1950 to present at a spatial resolution of approximately 9 km. In this study, two types of ERA5-Land reanalysis data were obtained from the C3S Climate Data Store (CDS): land surface temperature (LST) and volumetric soil moisture in the 0–7 cm layer. The temporal and spatial resolutions of these datasets are hourly and 0.1°, respectively. To align with other datasets, the hourly data were aggregated into daily values.

GLEAM is an algorithm framework that estimates different components of land evaporation and root-zone soil moisture based on satellite observations. GLEAM provides global-scale evaporation products at a spatial resolution of 0.25°, including transpiration, bare soil evaporation, open water evaporation, and sublimation. This study uses the updated version 3.8a of the GLEAM evaporation product released in 2023, which improves input data and algorithms compared to version 3.7 by incorporating MSWEP precipitation and ERA5 radiation data, and optimizing the use of vegetation optical depth. More information is available at the GLEAM website (https://www.gleam.eu/).

### 2.3. In Situ Soil Moisture Data

The International Soil Moisture Network (ISMN) is an open access global initiative dedicated to collecting and sharing in situ soil moisture measurements and related soil parameters. To date, ISMN has integrated data from 3030 ground stations across 77 monitoring networks, widely used for satellite product validation and the improvement of land surface, climate, and hydrological models. In this study, observational data from 58 ISMN stations during January–December 2019 were selected as a key validation dataset. These stations belong to three monitoring networks: Maqu, NGARI, and SMN-SDR. Additionally, data from 124 stations belonging to other monitoring networks were collected to enhance the spatial representativeness of the validation dataset, including Pali, Naqu, Saihanba, and SNOTE-China. Overall, soil moisture observations from 182 in situ stations were used as independent reference data for point-scale validation of the downscaled SM products. In selecting sites, this study prioritized networks with continuous, complete, and representative data covering major ecological and geomorphological types. The sites span the Tibetan Plateau, North China, and South China, across arid, semi-arid, temperate monsoon, and subtropical humid climates. A summary of these stations is provided in Table 2.

### 2.4. Other Auxiliary Products

Previous studies have shown that terrain plays a significant role in shaping the spatial distribution of soil moisture. Accordingly, this study incorporates the NASA SRTM DEM with a spatial resolution of 90 m as one of the predictors, covering more than 80% of the global land surface. In addition, soil texture strongly influences soil moisture by regulating processes such as infiltration rate, water retention, and permeability. The soil texture data used in this study are derived from the Harmonized World Soil Database version 2.0 (HWSD v2.0), which provides comprehensive information on global soil morphological, chemical, and physical properties at a resolution of approximately 1 km.

## 3. Methods and Evaluation Strategies

### 3.1. Data Preprocessing

In this study, high-resolution auxiliary data were mosaicked, clipped, and reprojected to the WGS-1984 geographic coordinate system, with invalid pixels removed based on quality control flags to eliminate cloud and noise interference. The data were resampled to 36 km and 1 km resolutions, where 36 km products were used with coarse-scale SMAP soil moisture for model training, and 1 km products served as inputs during prediction. Since the SMAP microwave sensor retrieves soil moisture from the top 0–5 cm layer, only in situ observations from corresponding depths were selected for validation. To unify the temporal resolution, hourly ERA5-Land data and ISMN soil moisture observations were aggregated to daily values using arithmetic averaging. The 16-day composite NDVI was assigned to each day within its compositing period, while the annual land cover product was treated as a static variable.

During model construction, pixel-wise MCCA SMAP soil moisture values and associated features were extracted as model inputs. These features included longitude, latitude, LST, evaporation, precipitation, albedo, NDVI, LCT, DEM, and soil texture (percentages of sand, silt, and clay). The missing and abnormal values in the MCCA SMAP SM data were systematically removed prior to training. All input data were normalized to a range of 0–1 and divided into training, validation, and testing sets at a ratio of 7:1.5:1.5, ensuring consistent data partitions across models. Based on the assumption of scale invariance between soil moisture and its auxiliary predictors, during the model prediction stage, high-resolution auxiliary data were resampled to a 1 km grid using bilinear interpolation for continuous variables(e.g., precipitation and evaporation), pixel averaging for LST, NDVI, and albedo, and majority voting for categorical variables such as land cover. The trained model was then applied to generate high spatial resolution downscaled SM products.

### 3.2. Methodology

**Figure 2:**
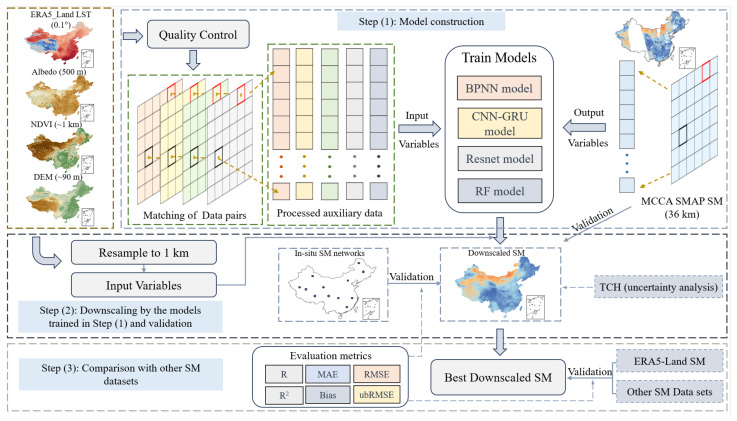
Flow chart of the downscaling framework of SM products used in this study.

Figure 2 illustrates the technical framework for constructing the downscaled SM product. First, MCCA SMAP soil moisture data and high-resolution auxiliary datasets were preprocessed, and valid data were selected through quality control. Next, a nonlinear relationship model between soil moisture and surface auxiliary parameters was established using coarse-resolution data to build the downscaling model. The trained model was then applied to high-resolution resampled auxiliary variables to generate the downscaled SM product. Finally, five quantitative metrics were used to comprehensively evaluate the performance of the downscaled products. This workflow encompasses the entire process from data preparation and model development to product validation. All procedures, including data preprocessing, model training, downscaling implementation, and result visualization, were conducted using MATLAB R2022a.

It should be noted that the downscaling algorithm itself does not inherently fill gaps in the original MCCA SMAP soil moisture data. The seamless downscaled results obtained in this study are mainly due to the fact that the 1 km resolution auxiliary datasets (e.g., LST, NDVI, LCT) used during prediction are themselves gap-free after resampling, resulting in a spatially continuous downscaled product.

#### 3.2.1. RF

**Figure 3:**
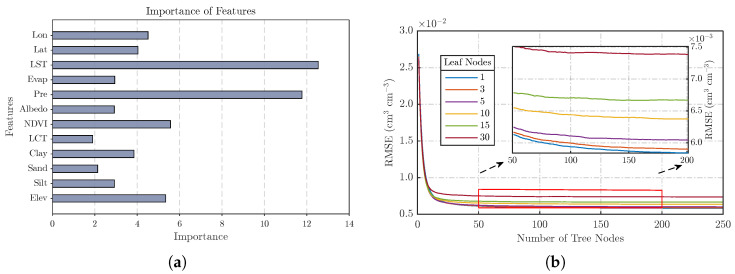
Construction of the RF model. The left panel shows the importance of selected features in predicting soil moisture during RF model construction, while the right panel displays the selection of optimal parameters for the RF model. (**a**) Feature importance in RF model. (**b**) Optimal parameter selection.

RF is a machine learning algorithm based on multiple decision trees and represents a typical Bagging approach in ensemble learning. Its core idea is to generate multiple training subsets through bootstrap sampling, train individual decision trees on each subset, and aggregate their predictions by averaging to obtain the final output, thereby improving accuracy and generalization ability. During node splitting, RF randomly selects a subset of features (or all features, depending on settings) for optimal partitioning, which helps prevent overfitting and enhances robustness against outliers and noise. Moreover, RF can assess the importance of each feature in predicting the target variable during training. The left panel of Figure 3 illustrates the importance of different features in the RF model, indicating that all selected variables contribute to soil moisture prediction, with LST and precipitation being the most influential. This is consistent with their physical roles: LST regulates evaporation, while precipitation affects SM through infiltration. In implementation, the RF downscaling model was developed on the MATLAB platform using the TreeBagger function. The out-of-bag (OOB) errors were recorded during training and used to assist hyperparameter selection. Model parameters were optimized using 10-fold cross-validation, yielding 150 decision trees and a minimum leaf sample size of 5, which ensured both predictive accuracy and efficiency.

#### 3.2.2. BPNN

Back Propagation Neural Network (BPNN) is a supervised learning algorithm applicable to both classification and regression tasks. It consists of an input layer, multiple hidden layers, and an output layer. The input layer receives external data, the hidden layers perform intermediate computations, and the output layer generates final predictions. Training involves two phases: forward propagation, where input data is processed layer by layer to produce outputs, and backpropagation, where the network’s weights and biases are adjusted based on output errors to improve performance. BPNN is well suited for learning complex nonlinear relationships by adjusting the number of hidden layers and neurons. In this study, the network architecture was optimized and set to four hidden layers with 20, 30, 30, and 33 neurons, respectively. All hidden layers used the tansig activation function, weights were updated with the Levenberg–Marquardt (trainlm) algorithm, and mean squared error (MSE) was employed as the loss function.

#### 3.2.3. CNN–GRU

Convolutional Neural Networks (CNN) extract spatial features through local connectivity and weight sharing, while Gated Recurrent Unit (GRU) models temporal dependencies using a gated mechanism. The CNN–GRU hybrid model proposed in this study operates as follows: (1) input sequences are divided into fixed-length subsequences; (2) spatial features are extracted through convolutional layers and downsampled via pooling layers, then mapped to fixed-dimensional feature sequences through fully connected layers; (3) the feature sequences are input into the GRU for temporal modeling, where the gated mechanism regulates information flow and captures long-term dependencies; (4) the GRU output is then mapped to the target prediction via a fully connected layer. In this study, the subsequence length *L* was optimized from candidate values {60,63,66,69,72,75} using the validation set. The convolution module comprised four 1D convolutional layers (kernel size 3×1, 64 channels, same padding), followed by batch normalization and ReLU activation, and a global max pooling layer for downsampling. The GRU hidden size was tuned within the same search range, with dropout (rate = 0.2) applied to mitigate overfitting.

#### 3.2.4. ResNet

**Figure 4:**
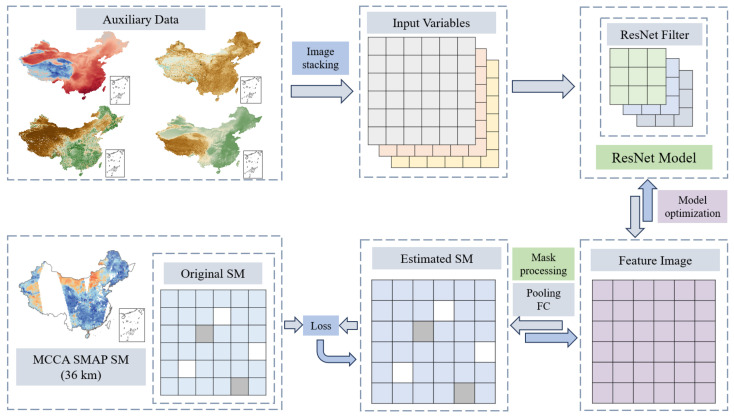
The construction process of the ResNet downscaling model.

Although the above models can effectively fit the complex nonlinear relationships between soil moisture and surface parameters through point-to-point training, they struggle to maintain spatial integrity. ResNet, proposed by He et al. [73], introduces residual block structures that alleviate the vanishing/exploding gradient problem in deep network, enabling deeper architectures to capture more complex nonlinear relationships. Based on this, a ResNet-based downscaling method is proposed in this study, with the specific workflow as follows: (1) stack the auxiliary data in a three-dimensional matrix of size w×h×n, where *h*, *w* and *n* represent latitude, longitude, and the number of auxiliary data types (n=10), respectively; (2) use multiple 3×3×n convolutional kernels to scan the matrix pixel by pixel and generate feature maps; (3) to address the issue of missing information in SMAP imagery, a soil moisture mask is generated based on the MCCA SMAP data. This mask is applied to the feature maps, followed by pooling and fully connected operations to obtain the estimated product; (4) the loss function is calculated based on the estimated product and the original MCCA data, and backpropagation is used to optimize the model. The detailed workflow is illustrated in Figure 4.

#### 3.2.5. Three-Cornered Hat Method

Given the limited number of in situ stations within the study area and the lack of reference data, this study employs the Three-cornered Hat (TCH) method to evaluate the uncertainty of four downscaled SM products. The TCH method, proposed by Tavella and Premoli [74], enables the assessment of uncertainty among different products of the same variable without requiring any prior knowledge. It has been widely used for uncertainty evaluation of GRACE products, evapotranspiration products, and SM products [75,76,77]. Compared to the Triple Collocation (TC) method, TCH allows the evaluation of three or more products and offers greater tolerance to error. The key steps of the TCH method are as follows:

Since the downscaled SM products obtained by different methods have the same spatial resolution, SM time series can be extracted for the same pixel, denoted as ${\left\{T_{i}\right\}}_{i=1,2,\ldots ,N}$, where *i* represents the ith SM product and *N* is the total number of products. The TCH method is then applied pixel by pixel to estimate the uncertainty of each type of downscaled product. At each pixel, the SM observation time series can be decomposed into two components: the true value $P_{\mathrm{true}}$ and an error term $\varepsilon_{i}$:

(1) $$T_{i}=P_{\mathrm{true}}+\varepsilon_{i},\;\forall i=1,2,\ldots ,N,$$

where $T_{i}$ is the time series of the ith SM product, $P_{\mathrm{true}}$ is defined as the true value of the SM time series, and $\varepsilon_{i}$ is the zero-mean white noise of the ith time series, which is orthogonal to $P_{\mathrm{true}}$. Then, the difference between each series and the reference series ($T_{r}$) is calculated using the following equation:

(2) $$Y_{i,r}=T_{i}-T_{r}=\varepsilon_{i}-\varepsilon_{r},\forall i=1,2,\ldots ,N-1,$$

where *Y* is a matrix with N−1 difference series. Since TCH is theoretically insensitive to the choice of $T_{r}$, RF downscaled SM is selected as $T_{r}$ in this study. The covariance matrix of *Y* can be represented by S=cov(Y).

Then, an N×N unknown covariance matrix *R* of the individual noises can be expressed as

(3) $$R=\left[\begin{array}{ccccc} r_{1,1} & r_{1,2} & \ldots & r_{1,(N-1)} & r_{1,N}\\ r_{2,1} & r_{2,2} & \ldots & r_{2,(N-1)} & r_{2,N}\\ \vdots & \vdots & \ddots & \vdots & \vdots \\ r_{(N-1),1} & r_{(N-1),2} & \ldots & r_{\left(N-1),(N-1\right)} & r_{(N-1),N}\\ r_{N,1} & r_{N,2} & \ldots & r_{N,(N-1)} & r_{N,N} \end{array}\right]\;,$$

where $r_{i,j}=r_{j,i}\left(i,j=1,2,\ldots ,N\right)$ is the covariance between the time series $\varepsilon_{i}$ and $\varepsilon_{j}$, and the diagonal elements $r_{ii}\left(i=1,2,\ldots ,N\right)$ are the unknowns that need to be determined in this study. To solve for the target unknowns, the matrix *R* is decomposed into submatrices related to the known *S* by the following equation:

(4) $$S=J\cdot R\cdot J^{T},J=\left[I-U\right],$$

where *I* is the (N−1)×(N−1) identity matrix, and *U* is the (N−1)×1 vector ${\left[1\;\;1\;\;\ldots \;\;1\right]}^{T}$. Since the number of unknown elements is larger than the number of equations, these target unknowns still cannot be solved based on Equation (4). The remaining elements require a reasonable way to obtain the unique value. To solve this problem, Tavella and Premoli [74] proposed a constrained minimization problem based on the Kuhn-Tucker theorem, with the objective function defined as:

(5) $$F_{\left(r_{1,N},\ldots ,r_{N,N}\right)}=\frac{1}{K^{2}}\sum_{i<j}^{N}r_{i,j}^{2},$$

and its constraint function is as follows:

(6) $$G\left(r_{1,N},\ldots ,r_{N,N}\right)=-\frac{H\left(r_{1,N},\ldots ,r_{N,N}\right)}{K}<0,$$

where $r_{1,N},\ldots ,r_{N,N}$ are free parameters, $K=\sqrt[{N-1}]{\det(S)}$ and det represents the determinant value of *S*. Since the matrix *R* is symmetric and has positive definiteness if and only if det(R)>0, *H* can be described as:

(7) $$H\left(r_{1,N},\ldots ,r_{N,N}\right)=\frac{\det(R)}{\det(S)}=r_{N,N}-\left[R-r_{N,N}\cdot U\right]S^{-1}{\left[R-r_{N,N}\cdot U\right]}^{T}>0.$$

Before the iterative computation, the initial values must satisfy the following constraint:

(8) $$r_{i,N}^{\left(0\right)}=0,i<N\;\mathrm{and}\;r_{N,N}^{\left(0\right)}={\left(2\cdot U^{T}\cdot S^{-1}\cdot U\right)}^{-1}.$$

After obtaining *N* free parameters by minimizing the objective function Equation (5), the remaining unknowns in *R* can be calculated using Equation (4), where the diagonal element ${\left\{r_{ii}\right\}}_{i=1,2,\ldots ,N}$ of *R* is the error variance of the time series ${\left\{T_{i}\right\}}_{i=1,2,\ldots ,N}$, and its square root result is the uncertainty of the corresponding SM product. In addition, the ratio of the uncertainty to the mean of the time series is the relative uncertainty.

### 3.3. Evaluation Metrics

The validation of downscaled SM products involves quantifying errors by comparing estimates with reference in situ measurements. To comprehensively evaluate product performance, five statistical metrics are used: Pearson correlation coefficient (*R*), bias (m^3^ m^−3^), mean absolute error (MAE, m^3^ m^−3^), root mean square error (RMSE, m^3^ m^−3^), and unbiased RMSE (ubRMSE, m^3^ m^−3^). The calculation formulas are as follows:

(9) $$Bias=E\left[\theta_{downscaled}\right]-E\left[\theta_{in\text{-}situ}\right],$$

(10) $$MAE=E\left[\left|\theta_{downscaled}-\theta_{in\text{-}situ}\right|\right],$$

(11) $$RMSE=\sqrt{E\left[{\left(\theta_{downscaled}-\theta_{in\text{-}situ}\right)}^{2}\right]},$$

(12) $$R=\frac{E\left[\left(\theta_{downscaled}-E\left[\theta_{downscaled}\right]\right)\left(\theta_{in\text{-}situ}-E\left[\theta_{in\text{-}situ}\right]\right)\right]}{\sigma_{downscaled}\sigma_{in\text{-}situ}},$$

(13) $$ubRMSE=\sqrt{E\left\{{\left[\left(\theta_{downscaled}-E\left[\theta_{downscaled}\right]\right)-\left(\theta_{in\text{-}situ}-E\left[\theta_{in\text{-}situ}\right]\right)\right]}^{2}\right\}},$$

where E[·] denotes the mean operator, $\theta_{downscaled}$ and $\theta_{in\text{-}situ}$ represent the downscaled and in situ soil moisture, $\sigma_{downscaled}$ and $\sigma_{in\text{-}situ}$ are their corresponding standard deviations. To ensure statistical robustness, the following criteria are applied: (1) validation sites with fewer than 50 valid observations are excluded; (2) only Pearson correlation coefficients with *p*-values < 0.05 are considered.

## 4. Results and Analysis

**Figure 5:**
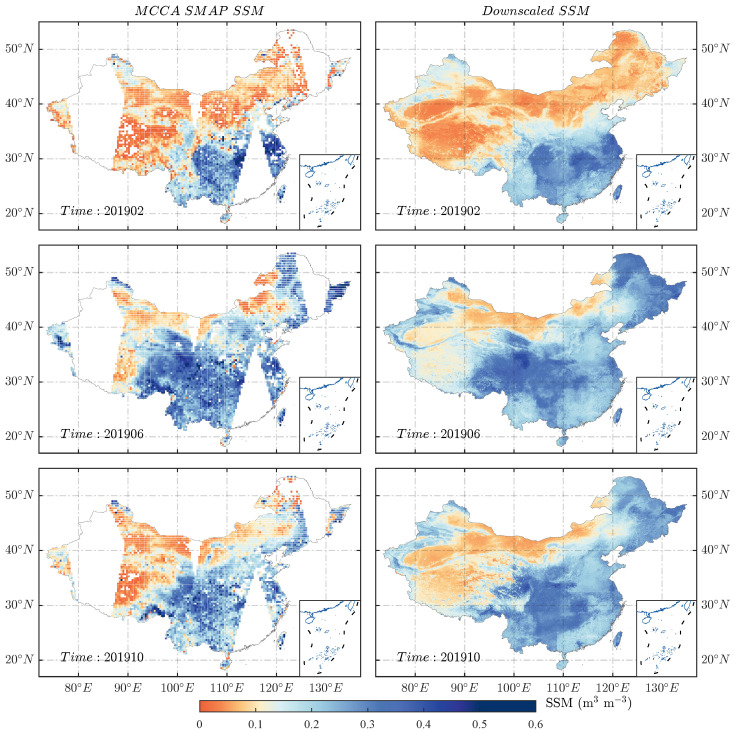
Comparison between the original MCCA SMAP SM map and the downscaled SM map ((**top**) to (**bottom**): 15 February, 15 June, and 17 October 2019).

Figure 5 compares the original MCCA SMAP SM data with gap-filled products on selected dates across different months. As shown, the downscaled SM products effectively fill spatial gaps while preserving the key characteristics of the original SMAP data—an essential improvement for analyzing spatiotemporal soil moisture patterns. Given the variation among downscaling methods, this section provides a comprehensive evaluation of each product’s performance from multiple perspectives.

### 4.1. Evaluation of the Models

**Figure 6:**
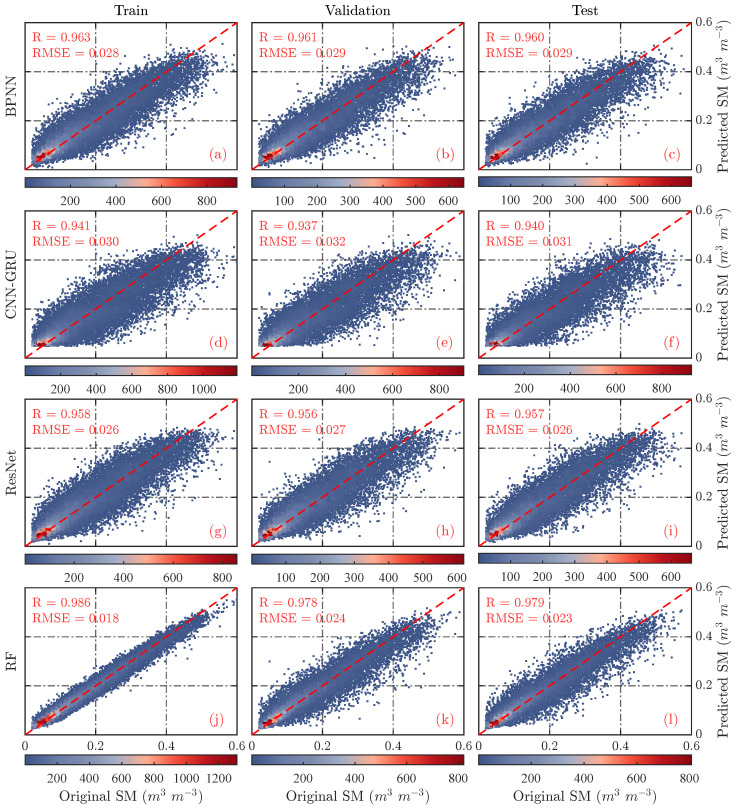
Scatter density comparison between SM predictions from different downscaling models and the original MCCA SMAP SM observations. Subfigures (**a**,**d**,**g**,**j**), (**b**,**e**,**h**,**k**), and (**c**,**f**,**i**,**l**) correspond to the training, validation, and testing sets, respectively, showing results from the BPNN, CNN–GRU, ResNet, and RF models.

To accurately assess the performance of different downscaling models, this section compares the predicted coarse-resolution SM values with the original MCCA SMAP SM observations (Figure 6). All models exhibited strong consistency with observations; aside from CNN–GRU on the validation and test sets, R values exceeded 0.94 and RMSEs were below 0.03 m^3^ m^−3^, indicating effective modeling of nonlinear relationships and accurate SM predictions. The consistency of evaluation metrics across subsets suggests no overfitting and reliable model outputs. Among the models, RF performs best, with R values above 0.97 and RMSEs below 0.025 m^3^ m^−3^ across all subsets. The other models yield similar performance, with R around 0.95 and RMSE near 0.03 m^3^ m^−3^.

**Figure 7:**
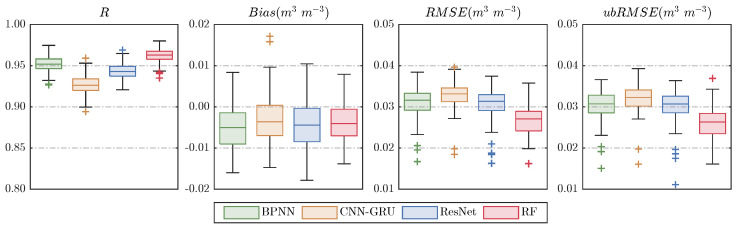
Boxplots of statistical metrics (R, Bias, RMSE, and ubRMSE) comparing original and downscaled SM products.

To better visualize these results, the distribution of daily evaluation metrics for each model is shown in Figure 7. In terms of R, RF exhibits the highest correlations, with values around 0.98 across the training, validation, and testing sets. BPNN performs second best, with R values consistently around 0.96 and a level of dispersion comparable to ResNet, though with slightly more noticeable outliers. ResNet follows, showing correlations around 0.95. CNN–GRU performs the weakest, with lower correlations near 0.94 and the largest dispersion among the four models. For bias, CNN–GRU, ResNet, and RF are similar, with median biases around −0.0036 m^3^ m^−3^, and RF showing the most concentrated distribution. BPNN performs slightly worse. Regarding RMSE and ubRMSE, RF significantly outperforms others, with median values of 0.027 m^3^ m^−3^ and 0.026 m^3^ m^−3^ respectively, and minimal variance. BPNN and ResNet show comparable performance, with median RMSE and ubRMSE of about 0.0315 and 0.0305 m^3^ m^−3^, respectively, while CNN–GRU performs the worst with medians of 0.033 and 0.032 m^3^ m^−3^. Overall, all four downscaling models perform well, with RF being the most robust and accurate.

### 4.2. Comparison of the Downscaled SM with In Situ Sites

**Figure 8:**
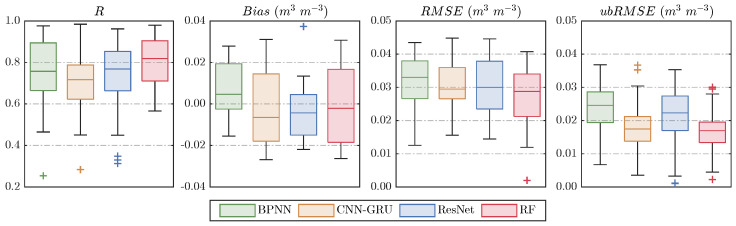
Boxplot of the distribution of statistical metrics (R, Bias, RMSE, and ubRMSE) between ground stations and downscaled results.

To quantitatively evaluate the performance of different downscaled SM products, this section uses in situ station data to validate their accuracy and reliability. Statistical metrics for each observation network are summarized in Table 3. Overall, all four downscaled products show relatively high correlations with in situ SM, particularly in the Pali, Naqu, Maqu, and Saihanba networks (R > 0.8), indicating that the variability of observed SM is well captured. In contrast, lower correlations are observed for SMN-SDR, likely due to its complex surface conditions and sparse station distribution. Among all methods, the RF product consistently maintains the highest or second-highest correlations across networks. Regarding bias, the four products exhibit small biases (<0.02 m^3^ m^−3^) in most networks, indicating minimal differences between predicted and observed values. The RF and ResNet products generally show lower and more stable biases. The CNN–GRU product exhibits the greatest bias fluctuation, with values ranging from a minimum of 0.005 m^3^ m^−3^ to a maximum of 0.021 m^3^ m^−3^. This is likely due to its structure: the GRU captures temporal dependencies while the CNN extracts spatial features, making the model sensitive to local deviations in auxiliary data. Variations in surface conditions and soil moisture dynamics across observation networks amplify these biases. Additionally, the ubRMSE values for the four products are similar across networks, indicating good overall accuracy, with RF showing slightly lower dispersion. Figure 8 further illustrates the distribution of evaluation metrics across all stations. The RF-based product exhibits the highest median R and the lowest bias, RMSE, and ubRMSE, with the most compact interquartile ranges, suggesting both higher accuracy and stronger robustness. In contrast, BPNN and CNN–GRU display broader metric distributions, reflecting less stable performance.

**Figure 9:**
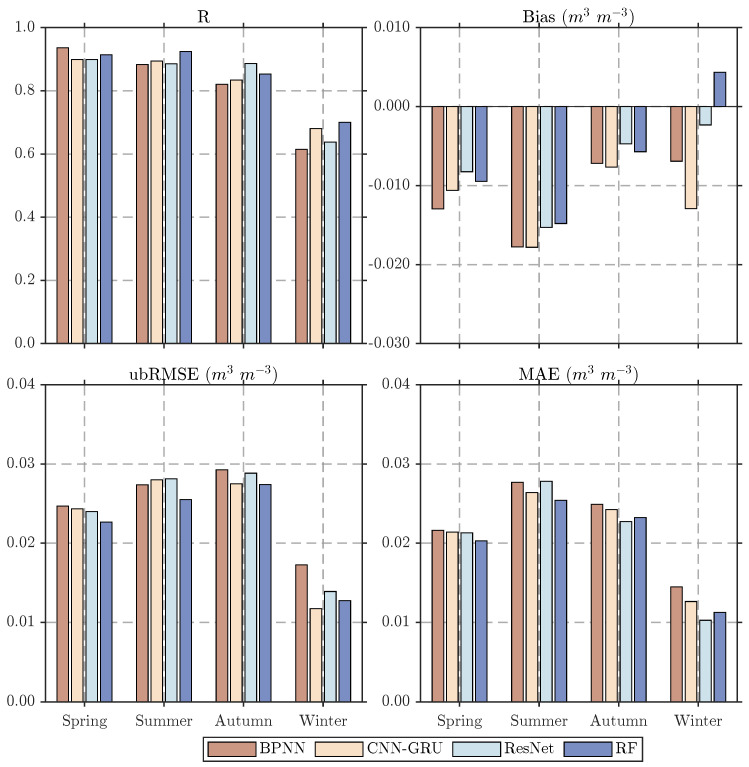
Seasonal comparison histogram of statistical metrics (R, Bias, ubRMSE, MAE) for different downscaled products at ground observation sites.

To evaluate the methods’ performance in different seasons, seasonal comparison analysis was conducted (Figure 9). The R-value histograms show that the correlation coefficient between all downscaled results and in situ observations is around 0.9 with little variation in spring and summer, around 0.86 in autumn, and significantly lower in winter. Bias analysis shows a general underestimation tendency during the warm seasons, particularly in summer, whereas winter biases are reduced. Compared with other models, RF exhibits more balanced seasonal behavior, with smaller bias magnitude in winter. This seasonal robustness of RF arises from its ensemble of decision trees, each trained on random subsets of samples and predictors, which reduces the impact of seasonal outliers while capturing the overall relationships among multiple predictors. The ubRMSE and MAE metrics show smaller seasonal differences, with generally lower values in winter, and RF consistently achieves the best or second-best performance in most seasons. Across seasons, the lower correlation in winter may result from frozen soil and increased microwave signal uncertainty, which hinder the models’ ability to capture subtle soil moisture variations. Meanwhile, low precipitation, reduced evaporation, and minimal vegetation activity lead to small overall soil moisture variability, allowing the models to predict average SM levels more accurately, and thus lower ubRMSE and MAE. Overall, the RF model demonstrates superior accuracy and stability across both observation networks and seasons, highlighting its stronger adaptability to diverse surface conditions and seasonal variability.

### 4.3. Spatio-Temporal Variation of the Downscaled SM

**Figure 10:**
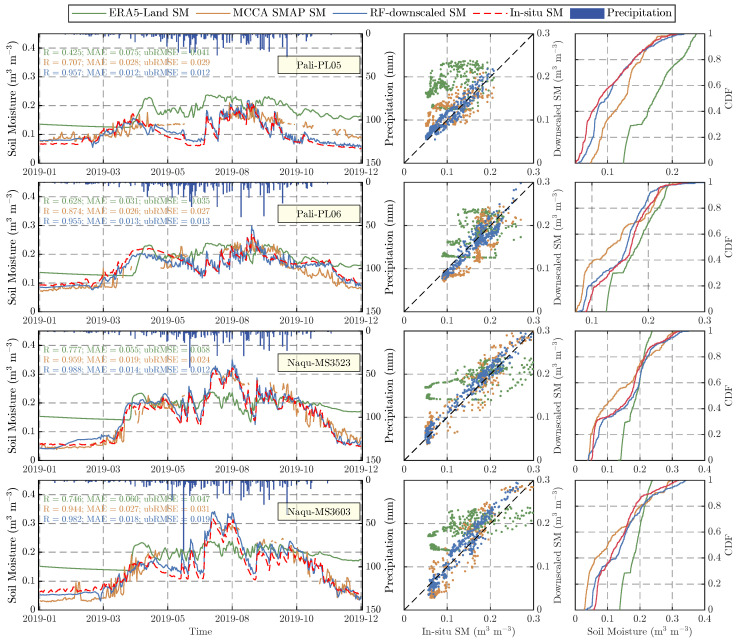
Temporal variations of ERA5-Land, MCCA SMAP, downscaled and insitu SM at four validation sites, with precipitation (blue bars), scatter plots versus in situ SM, and CDF curves shown in three columns.

To evaluate the capability of the RF-based downscaled SM product in capturing SM dynamics, this study conducted a comparative analysis among in situ observations, the MCCA SMAP SM product, the downscaled SM product, and the ERA5-Land reanalysis SM product at four randomly selected validation sites (Figure 10). The results indicate that all three datasets effectively captured the overall variation trend of SM. The time series shown in the first column reveal that the ERA5-Land product exhibited significant deviations from in situ observations during January–March and October–December, particularly overestimating SM at the MS3523 and MS3603 sites of the Naqu network (maximum bias: 0.13 m^3^ m^−3^) and showing notable overestimation of surface moisture (MAE = 0.075 m^3^ m^−3^) with poor dynamic representation at the PL05 site of the Pali network. In contrast, the downscaled SM product demonstrated higher accuracy and better agreement with measurements. Notably, precipitation (blue bars) showed a strong correlation with SM variations in the time series. The scatter plots in the second column indicate that downscaled SM data points were more tightly clustered around the 1:1 reference line, whereas MCCA SMAP SM displayed greater dispersion and ERA5-Land exhibited systematic over/underestimation. The cumulative distribution function (CDF) curves in the last column visually demonstrate that the downscaled SM distribution best approximates in situ observations. Comprehensive analysis confirms that the proposed downscaling framework can more accurately capture the dynamic characteristics of measured SM.

**Figure 11:**
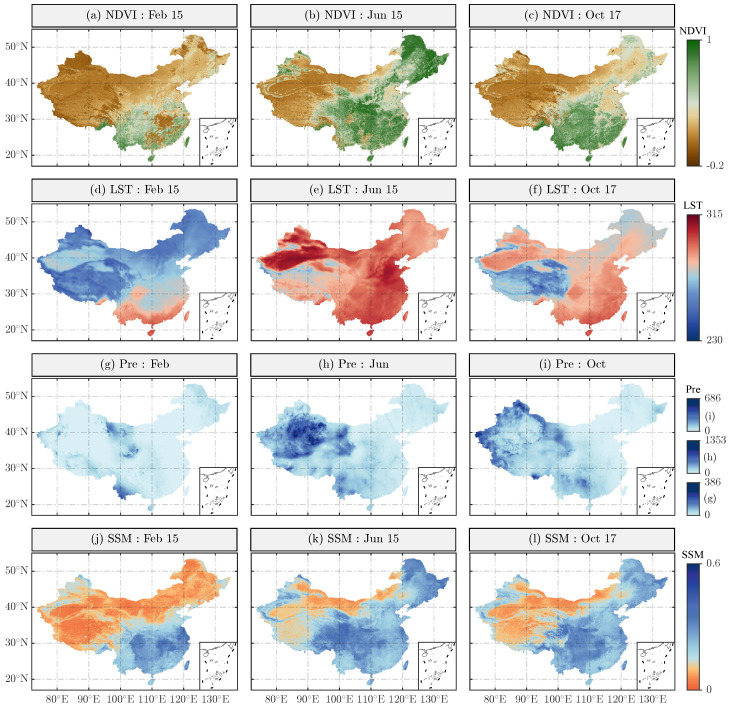
Spatial distributions of auxiliary variables (NDVI, Albedo, LST, and monthly total precipitation (Pre)) and downscaled SM (Feb 15, Jun 15, Oct 17)).

In addition, this study evaluated the performance of the downscaled SM product through spatial distribution maps. Since the spatial details of the downscaled SM mainly depend on high-resolution auxiliary variables, three random dates were selected to plot the spatial distributions of NDVI, LST, precipitation, and downscaled SM (Figure 11). The results show that NDVI values in the southeastern part of the study area were generally above 0.5, while those in the northwest were below 0.3, consistent with the higher vegetation coverage in the southeast. The LST on the Tibetan Plateau was significantly lower than in other regions, primarily due to the higher altitude and presence of permafrost, which also explains the lower SM values in this area. In northern regions, lower downscaled SM values corresponded with higher LST and NDVI values, reflecting the typical relationship between the LST-NDVI feature space and SM variability. However, this pattern was not observed in southern regions, which is related to their coastal location and the presence of major rivers such as the Yangtze River, also explaining why SM values there were significantly higher than in other areas. Given that elevation was identified as an important predictor of soil moisture comparable to NDVI (Figure 3), its influence can be further interpreted in conjunction with the topographic pattern shown in Figure 1. China exhibits a pronounced west–east descending terrain, characterized by dense river networks and enhanced moisture transport toward the eastern regions, which results in persistently higher soil moisture levels in eastern China compared with western regions. The analysis demonstrates that spatiotemporal variations of SM are influenced by multiple external factors, and the spatial consistency between auxiliary variables and downscaled SM confirms the importance of these input data for generating high-accuracy, spatiotemporally continuous SM products.

## 5. Discussion

### 5.1. Spatial Detail Representation of Downscaling Results and Model Limitations

**Figure 12:**
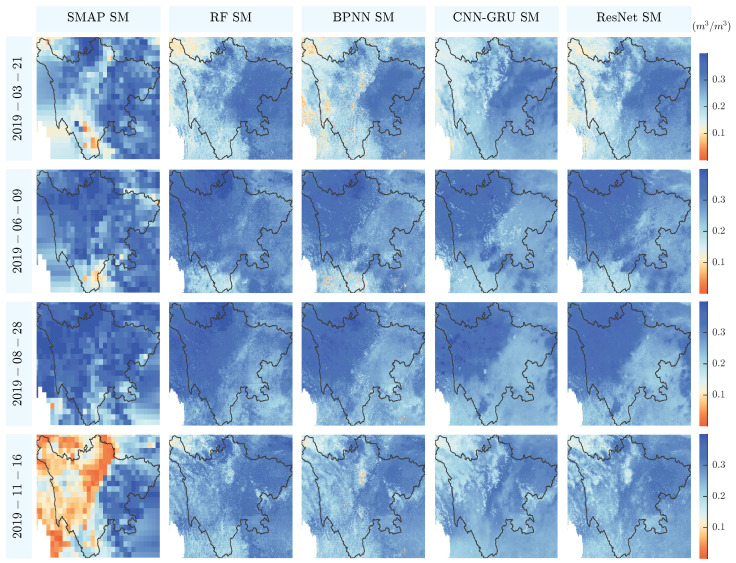
Spatial detail comparison of downscaled SM and the MCCA SMAP SM over Sichuan Province at different periods.

Figure 12 illustrates the downscaled SM products generated by RF, BPNN, CNN–GRU, and ResNet models compared with the MCCA SMAP product in Sichuan at multiple dates in 2019. Overall, all models improved the spatial resolution of SMAP products, revealing finer-scale heterogeneity in soil moisture distribution and filling data gaps caused by satellite orbit or observation limitations. This enhancement allows better representation of local features related to topography, land cover, and soil properties, which are otherwise indistinguishable in the coarse-resolution MCCA SMAP data. Among the models, RF generally preserves spatial gradients better and captures local variations more robustly, ResNet exhibits slightly smoothed outputs with occasional overestimation in high-SM areas, BPNN produces overly smooth fields, and CNN–GRU underestimates wet regions during certain periods. These differences reflect inherent disparities in model architecture and their sensitivity to local heterogeneity.

However, the image highlights several limitations. For example, on 16 November 2019, the original SMAP product shows low SM values in western Sichuan and high SM values in the east, whereas all four downscaling models fail to reproduce this pronounced contrast. This suggests that the models could not fully capture fine-scale spatial variations in regions with highly heterogeneous soil moisture. Possible reasons include: (1) model limitations in learning subtle spatial gradients due to the complexity of nonlinear relationships between SM and auxiliary variables, especially under high heterogeneity, and (2) inherent limitations of the MCCA SMAP product, which relies on a single satellite source, potentially constraining the representation of extreme or localized SM patterns.

It should be noted that this analysis is constrained by observational limitations. High-density in situ soil moisture observations covering the entire study area—especially the high-altitude, complex terrain of the Tibetan Plateau and some remote regions of Sichuan—are currently lacking. This data limitation represents a major constraint of this study and restricts in-depth discussion of the physical consistency of small-scale spatial structures. Future research could incorporate additional satellite SM products, such as SMOS or AMSR2, and integrate more ground observations to improve spatial fidelity and reduce uncertainty.

### 5.2. Uncertainty Analysis of the Downscaling Results

**Figure 13:**
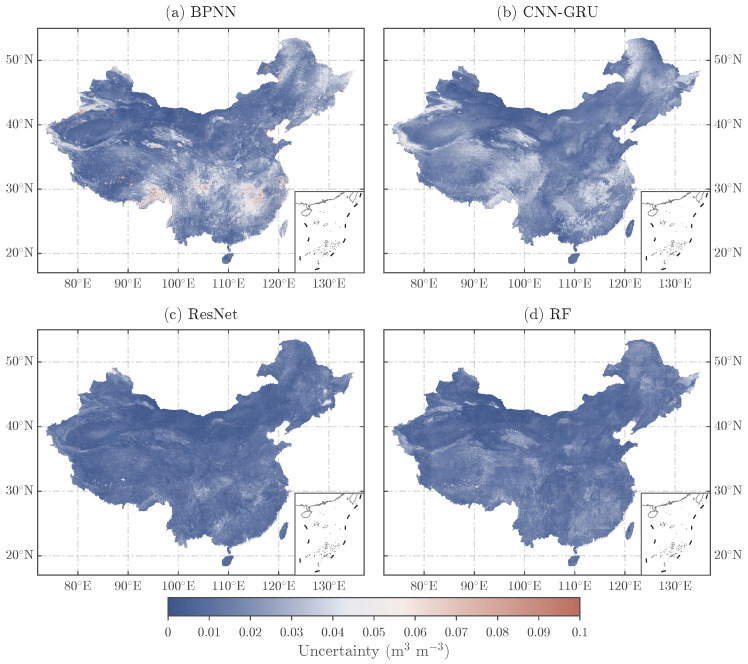
Comparison of uncertainty results for four downscaled SM products based on TCH.

To address the limitations of ground-based validation, the TCH method was employed to quantify the spatial uncertainty of the four downscaled products across the study area (Figure 13). The results show that the ResNet- and RF-based products exhibit relatively low uncertainty (<0.02 m^3^ m^−3^) in most regions, but higher uncertainty (around 0.04 m^3^ m^−3^) is observed in the Tibetan Plateau and the northeastern edge regions. A comparison of the original SM map and the downscaled products reveals significant SM variations in these regions, possibly due to complex land cover and topographic features. However, these areas lack a dense in situ monitoring network, which greatly limits the study of SM variation patterns in regions with abrupt topographic changes. Increasing monitoring stations in relatively accessible low-elevation and flat areas surrounding these high-uncertainty regions could help improve spatial representativeness and provide more reliable reference data for model evaluation and development. In contrast, the BPNN and CNN–GRU models show significantly higher uncertainty in the Tibetan Plateau, the northwestern edge regions, and the northeastern edge regions, with the BPNN model exhibiting particularly high uncertainty, indicating considerable deviation between its predictions and the actual values. Based on the earlier analysis, the RF-based downscaled SM product performs the best and is regarded as the most reliable.

## 6. Conclusions

High-precision and high-resolution soil moisture information is essential for understanding land–atmosphere interactions and hydrological processes over China. This study applied four machine learning-based downscaling models (RF, BPNN, ResNet, and CNN–GRU), together with multi-source auxiliary variables, to enhance the spatial resolution of the MCCA SMAP SM product from 36 km to 1 km across China. Overall results demonstrate that all four models substantially improve the spatial detail and continuity of the original MCCA SMAP SM product, enabling a more refined representation of SM heterogeneity under diverse climatic and land surface conditions in China. Among all models, the RF exhibits the best overall performance. Compared with in situ observations, the RF product shows generally higher correlations and lower biases, while the other downscaled products perform relatively less well. In addition, time-series analyses against ERA5-Land and in situ measurements indicate that the RF downscaled product accurately captures the temporal variations observed at ground stations, with a higher level of consistency than the other products. Uncertainty analysis based on the TCH method reveals generally lower uncertainty for RF and ResNet products, while higher uncertainty persists in regions with complex terrain and sparse ground observations, such as the Tibetan Plateau and marginal northeastern areas. It should be noted that this study is subject to several limitations. First, the validation of fine-scale spatial details is constrained by the uneven and sparse distribution of ground-based SM observations, particularly in mountainous and high-altitude regions. Meanwhile, the absence of a dense nationwide monitoring network means that the reliability of downscaled results in areas without in situ measurements relies primarily on the model’s generalization capability, which inevitably limits the assessment of product performance at the national scale. Second, the downscaling framework relies on a single satellite SM product (MCCA SMAP), which may limit the representation of localized or extreme SM variability under highly heterogeneous surface conditions. Although the downscaled maps cover island regions such as Hainan and Taiwan, their accuracy could not be quantitatively assessed due to the lack of available in situ observations.

Compared to traditional downscaling methods, machine learning demonstrates distinct advantages in establishing nonlinear relationships between soil moisture and surface auxiliary parameters. Our future work will focus on the following two aspects: (1) integrating multi-source satellite soil moisture products (e.g., SMOS and AMSR2) to reduce the uncertainty associated with reliance on a single SM dataset and to better characterize soil moisture variability under heterogeneous surface conditions; and (2) incorporating additional surface descriptors, such as surface roughness and vegetation structure, together with improved ground observation coverage, to further enhance the robustness and reliability of machine learning-based downscaling frameworks for representing spatiotemporal soil moisture dynamics over China. In addition, future studies will place greater emphasis on soil moisture downscaling and accuracy validation across diverse landscape types in China, including mountainous, plain, and island regions, as more in situ measurements become available.

## Figures and Tables

**Table 1:** Summary of the data sets used in this study.

Product Name	Variable(s)	Spatial Resolution	Temporal Resolution	Units	Reference
MOD13A2 v6.1	Vegetation Index	1 km	16 days	-	[53]
MCD12Q1 v6.1	Land Cover Type	500 m	yearly	-	[54]
MCD43A3 v6.1	Surface Albedo	500 m	daily	-	[55]
MCCA SMAP v1	SM	36 km	daily	m^3^ m^−3^	[56]
GPM	Precipitation	0.1°	daily	mm/day	[57]
ERA5-Land	SM & LST	0.1°	hourly	m^3^ m^−3^ (K)	[20]
GLEAM v3.8a	Evaporation	0.25°	daily	m^3^ m^−3^	[58]
ISMN	In situ SM	Points	hourly	m^3^ m^−3^	[59]
SRTM	DEM	90 m	-	m	[60]
HWSD v2.0	Soil Texture	1 km	-	-	[61]

**Table 2:** The descriptions of in situ soil moisture networks used in this study.

SM Network	No. of Sites	Sensor	Measure Depths (cm)	Reference
Pali	25	EC-5TM	5, 10, 20, 40	[64,65]
Naqu	57	EC-5TM	5, 10, 20, 40	[64,66,67]
Maqu	12	EC-5TM	5, 10, 20, 40, 80	[68]
NGARI	12	EC-5TM	5, 10, 20, 40, 80	[69]
Saihanba	25	EC-5TM, XST	5, 10	[70]
SMN-SDR	34	EC-5TM	3, 5, 10, 20, 50	[17,71]
SNOTE-China	17	Meter 5TM	5, 10, 20, 40	[72]

**Table 3:** Statistical results of evaluation metrics between different downscaled SM products and each in situ observation network.

Metrics	R (*p*-Values < 0.05)				Bias (m^3^m^−3^)				ubRMSE (m^3^m^−3^)			
Networks	BPNN	CNN–GRU	ResNet	RF	BPNN	CNN–GRU	ResNet	RF	BPNN	CNN–GRU	ResNet	RF
Pali	0.920	0.893	0.903	0.935	−0.012	−0.012	−0.008	−0.005	0.020	0.019	0.022	0.018
Naqu	0.812	0.785	0.890	0.917	0.011	−0.017	−0.015	0.006	0.024	0.020	0.017	0.017
Maqu	0.893	0.791	0.898	0.897	0.013	0.005	−0.013	−0.011	0.021	0.017	0.020	0.015
NGARI	0.709	0.693	0.786	0.779	0.017	−0.019	−0.017	−0.014	0.025	0.019	0.021	0.016
Saihanba	0.787	0.719	0.731	0.882	0.016	0.021	−0.019	0.012	0.026	0.020	0.027	0.019
SMN-SDR	0.664	0.659	0.668	0.763	−0.017	0.017	−0.010	0.009	0.027	0.022	0.025	0.023
SNOTE-China	0.745	0.728	0.752	0.791	0.012	−0.013	−0.012	0.011	0.021	0.015	0.022	0.017

## Data Availability

The National Aeronautics and Space Administration for providing the SMAP soil moisture data (https://nsidc.org/data/spl2smp/versions/8 (accessed on 15 March 2024)), the Moderate Resolution Imaging Spectroradiometer (MODIS) for providing the MODIS data (https://lpdaac.usgs.gov/data/ (accessed on 20 March 2024)), NASA’s Global Precipitation Measurement (GPM) Mission for providing the precipitation data (https://gpm.nasa.gov/data/directory (accessed on 25 March 2024)), the European Centre for Medium-Range Weather Forecasts (ECMWF) for providing the ERA5-Land reanalysis data (https://cds.climate.copernicus.eu/datasets/reanalysis-era5-land (accessed on 28 March 2024)), the Global Land Evaporation Amsterdam Model (GLEAM) team for providing the evaporation data (https://www.gleam.eu/ (accessed on 5 April 2024)), the International Soil Moisture Network (ISMN) for providing the in situ soil moisture data (https://ismn.earth/en/ (accessed on 5 December 2024)), NASA’s Shuttle Radar Topography Mission (SRTM) for providing the DEM data (https://www.earthdata.nasa.gov/sensors/srtm (accessed on 10 April 2024)), and the Harmonized World Soil Database (HWSD) for providing the soil property data (https://gaez.fao.org/pages/hwsd (accessed on 12 April 2024)). The author also thanks the National Tibetan Plateau/Third Pole Environment Data Center (http://data.tpdc.ac.cn (accessed on 15 October 2024)) for providing the MCCA SMAP dataset as well as the Pali and Naqu in situ soil moisture observation data.
